# Growth performance, caecal microbiome profile, short-chain fatty acids, and litter characteristics in response to placement on reused litter and combined threonine, arginine and glutamine supplementation to juvenile male broiler chickens

**DOI:** 10.1186/s42523-023-00240-0

**Published:** 2023-03-22

**Authors:** Marwa A. Hussein, Farina Khattak, Lonneke Vervelde, Spiridoula Athanasiadou, Jos G. M. Houdijk

**Affiliations:** 1 Monogastric Science Research Centre, Scotland’s Rural College (SRUC), Edinburgh, UK; 2 The Roslin Institute and Royal (Dick) School of Veterinary Studies, University of Edinburgh, Edinburgh, UK; 3 Nutrition and Nutritional Deficiency Diseases Department, Faculty of Veterinary Medicine, Mansoura University, Mansoura, Egypt; 4 Animal and Veterinary Sciences, Scotland’s Rural College (SRUC), Edinburgh, UK

**Keywords:** Amino acids, Reused litter, Ideal protein, Broilers, Growth performance, Caecal microbiome, Litter characteristics

## Abstract

**Background:**

Exposure of broilers to litter microbiome may increase specific amino acid (AA) requirements towards activated immune responses. This may challenge the generality of the ideal protein (IP) concept, in which dietary essential AA to lysine ratios aimed to mimic presumably constant AA to lysine ratios in whole bird requirements. Therefore, we tested the effect of threonine, arginine and glutamine (TAG) supplementation to IP-based control diets (C) on performance, caecal microbiome composition, short-chain fatty acids and litter characteristics of broiler chickens placed on reused litter.

**Results:**

Thirty-two pens with ten male broiler chickens each were used in a 2 × 2 factorial arrangement of two diet treatments (with or without TAG supplementation) and two litter treatments (placement on clean or reused litter) for 21 days (n = 8). Caecal contents were analysed for microbiome profile using percent guanine + cytosine (%G + C profile) method and short chain fatty acids. TAG-supplemented birds underperformed compared to C birds (*P* = 0.002), whereas birds placed on reused litter outperformed those on clean litter (*P* = 0.047). Diet, reused litter and their interaction impacted the %G + C profile at different ranges. Whilst TAG supplementation reduced bacterial abundance at %G + C 51–56 (*P* < 0.05), reused litter placement tended to reduce %G + C 23–31 and increase %G + C 56–59 (*P* < 0.10). However, TAG supplementation reduced bacterial abundance at %G + C 47–51 (*P* < 0.05) and increased caecal branched chain fatty acids on clean litter only (*P* = 0.025). Greater levels of propionic acid were observed for C birds placed on reused litter only (*P* = 0.008). Litter pH was greater for reused litter pens than clean litter pens at day 21 (*P* < 0.001). In addition, litter moisture content was less for TAG birds and reused litter pens compared to C birds (*P* = 0.041) and clean litter pens (*P* < 0.001), respectively.

**Conclusions:**

These data support the view that irrespective of performance benefits arising from bird placement on reused litter, TAG supplementation to IP-formulated baseline rations impaired growth, supported by the lowered abundance of caecal bacteria known to dominate in well-performing birds and greater levels of caecal branched chain fatty acids.

## Introduction

Dietary protein requirement should represent the sum of a balanced level of essential amino acids (AA) and sufficient non-essential AA that fulfils age-dependant AA requirement for optimal performance. This view is the basis for the ideal protein (IP) concept that has been introduced into diet formulation for poultry to optimise AA supply and, thus, nitrogen utilisation [1–3]. The IP concept represents dietary essential AA (eAA) to lysine ratios as in whole bird AA requirements for maintenance and production, leading to an ideal AA profile in which all eAA are equally limiting [4, 5]. Several studies reported a positive impact of AA supplementation on performance, e.g. threonine (Thr) [6–8], arginine (Arg) [9–11], but also the non-essential amino acid glutamine (Gln) [12–14]. However, AA supplementation to an IP formulated baseline would not be expected to improve performance but result in excess AA intake. The latter would be expected to facilitate proteolytic activity of the hindgut microbiome resulting in changes in composition and/or metabolite production, deteriorated litter quality and potentially reduced performance [15]. However, birds under external microbial and other pathogen exposure would be expected to require different dietary AA ratios for the combined optimal performance and enhanced immune responses, arising from competition for limiting AA between these functions [16–19], which may thus challenge the generality of the IP concept.

Each of Thr, Arg and Gln has been implicated in host responses to viral, bacterial and/ or parasitic exposure. For instance, Thr plays a vital role in the maintenance of intestinal barrier integrity and mucin synthesis [8, 20] and is a major component of gamma globulin [21, 22]. Supplementation with Thr has been shown to increase hemagglutination titres of birds infected with the Newcastle disease virus [23]. In addition, Thr supplementation has been found to improve gut health during salmonellosis in broilers [24]. The supplementation with Arg, which is a precursor for nitric oxide, polyamines, and creatine [25], has been shown to improve intestinal morphology [26] during sub-clinical enteric challenges as it can ameliorate coccidiosis-induced intestinal villus damage and goblet cell depletion [27]. As the main energy source for immune and intestinal epithelial cells, Gln could become limiting during elevated Gln requirements arising from enteric challenges [28, 29]. Indeed, enhanced growth performance during sub-clinical challenges upon Gln supplementation has been shown to concur with improved gut morphology, i.e., increased villus height and lowered crypt depth, which improves absorptive capacity [30, 31]. Wu et al. [14] further reported that Gln supplementation may ameliorate detrimental effects of *Salmonella enteritidis* infection on intestinal immune barrier functions and lymphoid organ weights (i.e., bursa of Fabricius, spleen and thymus).

Host responses to specific AA supplementation may be sensitive to the level of microbiota exposure, as immune and possible pathological responses could result in increased whole bird AA requirements that deviate from the ideal AA profile. In the current study, placement on reused litter, which may impact caecal microbiome characteristics [32], was used to create two contrasting levels of microbiota exposure, under which the AA supplementation was tested. We hypothesised that the effect of Thr, Arg and Gln (TAG) supplementation to IP formulated basal rations on broiler growth performance, caecal microbiome parameters, and litter characteristics (pH and moisture content) is sensitive to reused litter exposure. To our knowledge, this is the first time that the effects of AA supplementation to IP-based rations are assessed on caecal microbiome composition and fermentation metabolites of broilers placed on reused litter.

## Results

### Diet analysis and growth performance

Although relative to the TAG diets, the non-TAG AA levels of the C diets were on average 4% greater and 3% smaller for the starter and grower phase, respectively, overall, the analysed nutrient and individual AA composition of the experimental diets were within the expected range (Table 1). Since ~ 98.5% of the diets were derived from a common basal, and the part that varied consisted of starch or TAG only, observed variation likely reflects variation in analysis rather than an actual chemical composition. The effect of TAG supplementation and litter treatments on growth performance during the entire growth phase (days 0–21) is shown in Table 2. There were no significant interactions between diet and litter treatment on performance data. However, diet treatment impacted growth performance measurements, i.e., body weight gain (BWG), feed intake (FI) and crude protein conversion (CPC), as TAG birds had smaller BWG, FI and larger CPC than C birds. Furthermore, birds placed on reused litter had greater BWG and tended to have better feed conversion ratio (FCR) and CPC than birds placed on clean litter. Mortality was low at 0.3% (1 out of 320 birds placed). This 11-day-old bird was culled due to hunched posture. The post-mortem reported that there was a large yolk sac remnant with necrotic content.

**Table 1:** Analysed chemical composition, gross energy, and total amino acid content of the experimental starter (0 to 11 days) and grower (11 to 21 days) rations

	Starter rations		Grower rations	
	C	TAG	C	TAG
*Chemical composition*				
DM (%)	88.00	89.00	88.30	88.30
Crude ash (%)	6.20	10.40	5.70	5.30
CP (%)	22.82	24.12	21.14	23.21
NDF (%)	7.80	7.90	7.70	7.50
ADF (%)	4.15	3.88	3.53	3.48
Sucrose (%)	5.36	3.95	4.17	4.44
Total starch (%)	35.80	34.10	39.80	36.00
EE (%)	3.83	4.21	4.80	4.53
AHEE (%)	4.74	5.02	5.74	5.24
GE (MJ/kg)	16.45	16.63	16.77	16.77
*Amino acids composition (%)*				
Methionine	0.55	0.50	0.46	0.50
Cysteine	0.36	0.36	0.35	0.35
Methionine + cysteine	0.90	0.86	0.81	0.85
Lysine	1.45	1.31	1.26	1.35
Threonine	0.98	1.16	0.87	1.10
Arginine	1.48	1.76	1.36	1.68
Isoleucine	0.95	0.90	0.88	0.90
Leucine	1.55	1.49	1.47	1.50
Valine	1.10	1.02	0.99	1.02
Histidine	0.52	0.50	0.50	0.51
Phenylalanine	1.02	0.98	0.97	1.00
Glycine	0.87	0.85	0.84	0.84
Serine	1.04	1.02	0.99	1.01
Proline	1.33	1.33	1.27	1.33
Alanine	0.88	0.85	0.84	0.86
Aspartic acid	2.04	1.95	1.91	1.97
Glutamic acid	4.31	5.19	4.17	5.16

*C* Control; *TAG* Threonine, arginine and glutamine supplemented diets; *DM* Dry matter; *CP* Crude protein; *EE* Ether extract; *AHEE* Ether extract preceded by acid hydrolysis; *NDF* Neutral detergent fibre; *ADF* Acid detergent fibre; *GE* Gross energy

**Table 2:** Growth performance of broilers fed C or TAG diets and placed as day-old on either clean or reused litter over 21 days

Litter	Diet	BWG, g	FI, g	FCR, g/g	CPC*
Clean	C	738	1044	1.447	0.298
	TAG	663	914	1.417	0.319
Reused	C	779	1028	1.349	0.278
	TAG	708	947	1.371	0.308
	SED	28.8	21.7	0.051	0.011
*Means for main effect of litter*					
Clean		700^a^	979	1.432	0.308
Reused		743^b^	988	1.360	0.293
	SED	20.4	15.4	0.036	0.008
*Means for main effect of diet*					
	C	758^b^	1036^b^	1.398	0.288^a^
	TAG	685^a^	931^a^	1.394	0.313^b^
	SED	20.4	15.4	0.036	0.008
*P-values for main effects and interaction*					
Litter		0.047	0.581	0.056	0.068
Diet		0.002	< 0.001	0.902	0.004
Litter × diet		0.924	0.134	0.483	0.544

*BWG* Body weight gain; *FI* Feed intake; *FCR* Feed conversion ratio; CPC* Crude protein conversion = FI (kg) × CP content diet (g/kg)/BWG (g); *C* Control diets; *TAG* Threonine, arginine and glutamine supplemented diets; *SED* Standard error of difference; Means within the same column with different superscripts differ at *P* < 0.05; Simple means represent 8 pens of 10 birds per pen

### Caecal %G + C profile

**Fig. 1:**
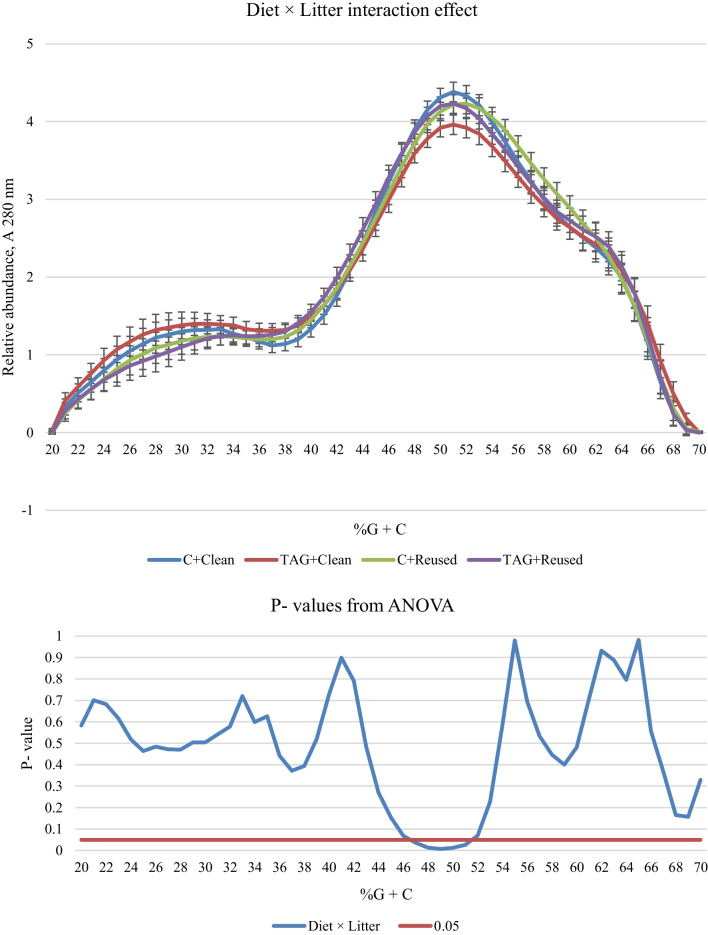
Diet and Litter interaction effects on the %G + C profile of caecal bacteria from broilers aged 21 days. In the upper panel, the solid blue line represents the mean %G + C profile of birds fed C diets and placed on clean litter, the solid red line represents the mean %G + C profile of birds fed TAG diets and placed on clean litter, the solid green line shows the mean %G + C of birds fed C diets and placed on reused litter and the solid purple line illustrates the mean %G + C of birds fed TAG diets and placed on reused litter (n = 8). In the lower panel, the solid blue line shows the results from ANOVA and the solid red line marks the threshold of *P* = 0.05

**Fig. 2:**
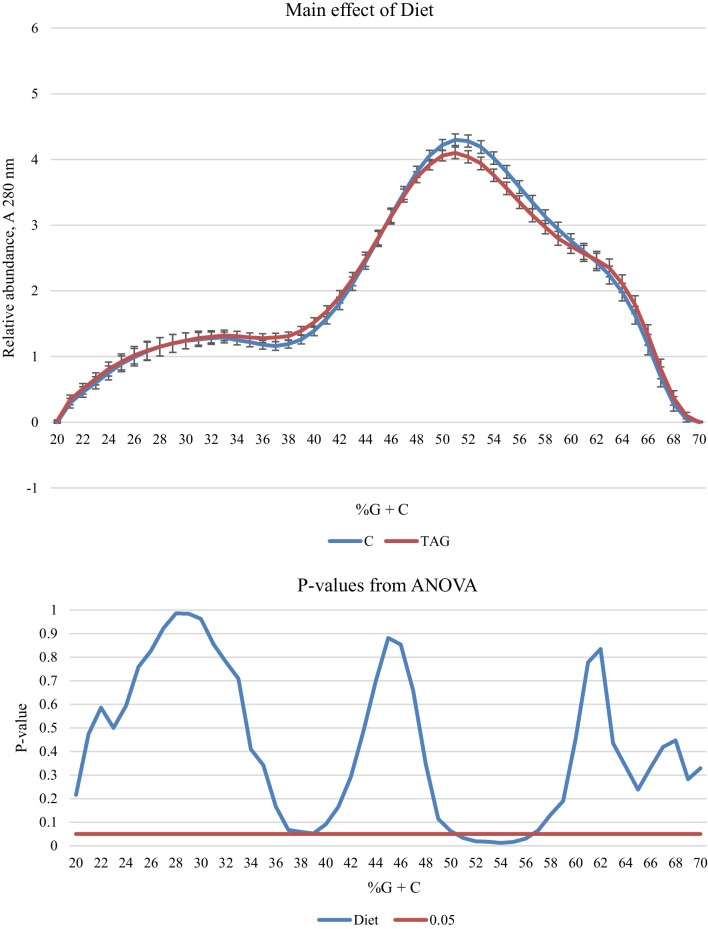
The main effect of Diet treatment on the %G + C profile of caecal bacteria from broilers aged 21 days. In the upper panel, the solid blue line represents the mean %G + C profile of birds fed C diets and the solid red line shows the mean %G + C profile of birds fed TAG diets (n = 16). In the lower panel, the solid blue line shows the results from ANOVA and the solid red line marks the threshold of *P* = 0.05

**Fig. 3:**
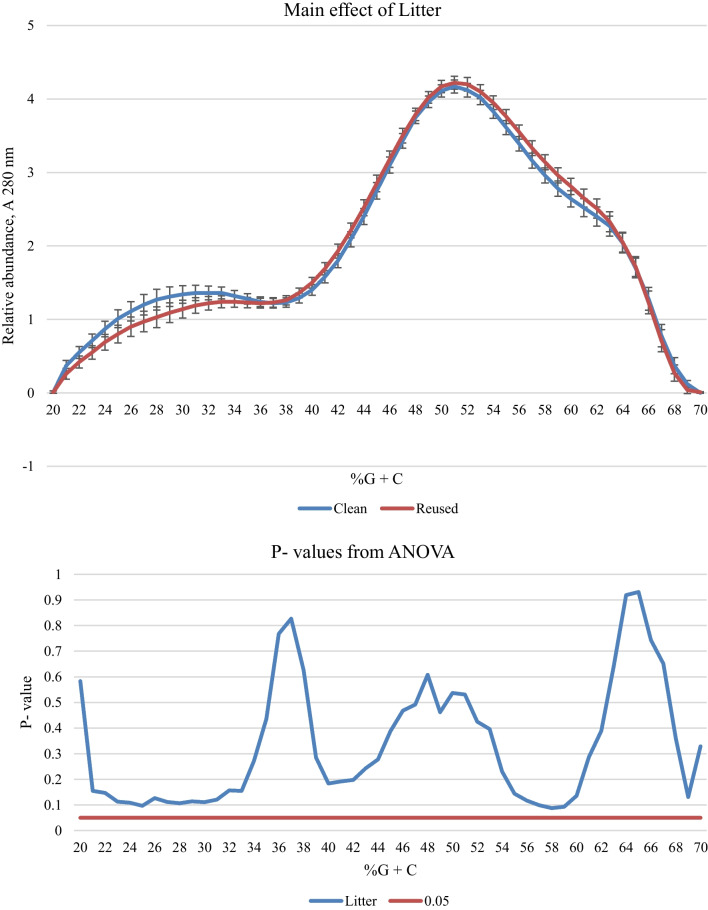
The main effect of Litter treatment on the %G + C profile of caecal bacteria from broilers aged 21 days. In the upper panel, the solid blue line represents the mean %G + C profile of birds placed on clean litter and the solid red line represents the mean %G + C profile of birds placed on reused litter (n = 16). In the lower panel, the solid blue line shows the results from ANOVA and the solid red line marks the threshold of *P* = 0.05

The percent guanine + cytosine (%G + C) profile of the total chromosomal DNA was determined to illustrate the relative abundance of the entire microbial community as a response to diet or litter treatment and thus enables the detection of any putative alterations at the community level. Diet and litter treatment interacted for caecal %G + C 47–51 (*P* < 0.01) as birds fed the test diet (TAG) compared to birds fed the control diet (C) showed a lower abundance over that range but only when placed on clean litter (Fig. 1). However, diet treatment affected %G + C at a higher range, as TAG birds displayed a significant shift towards a lower abundance of bacteria at %G + C 51–56 than C birds (Fig. 2). In contrast, litter treatment did not significantly affect %G + C profile; the consistently lower %G + C 23–31 and greater %G + C 56–59 for birds on reused litter compared to those on clean litter averaged at *P* = 0.112 and *P* = 0.099, respectively (Fig. 3).

### Caecal SCFA concentration and composition

The total short chain fatty acids (SCFA) concentration in the caecal content and its composition were determined as an indicator of the fermentative activity of the microbial population and are presented in Table 3. Total SCFA concentration did not significantly differ between treatments. However, a significant interaction between diet and litter treatments was observed for the percentage of propionic acid and branched-chain fatty acids (BCFA); the latter consisted of iso-butyric acid only, as the other two BCFA (2-methyl-butyric acid and iso*-*valeric acid) were below the detection limit. C birds had a greater percentage of propionic acid than TAG birds on reused litter only. In addition, whilst TAG birds had a greater proportion of iso-butyric acid than C birds across litter treatments, the interaction indicated this was most pronounced on clean litter.

**Table 3:** Short chain fatty acids in the ceca of 21-day old broilers fed C or TAG diets and placed on either clean or reused litter

Litter	Diet	Total SCFA (mM)	%Acetic acid	%Propionic acid	%Butyric acid	%Lactic acid	%Iso-butyric acid
Clean	C	105.10	72.45	3.98^a^	13.34	10.07	0.10^a^
	TAG	97.60	71.32	5.67^ab^	12.88	9.79	0.32^b^
Reused	C	92.20	73.99	6.35^b^	10.45	9.08	0.12^a^
	TAG	92.40	72.26	3.65^a^	11.79	12.14	0.16^a^
	SED	8.15	2.06	1.05	1.55	1.80	0.05
*Means for main effect of litter*							
Clean		101.40	71.88	4.83	13.11	9.93	0.21
Reused		92.30	73.13	5.00	11.12	10.61	0.14
	SED	5.77	1.46	0.74	1.09	1.27	0.04
*Means for main effect of diet*							
	C	98.60	73.22	5.16	11.89	9.57	0.11^a^
	TAG	95.00	71.79	4.66	12.33	10.97	0.24^b^
	SED	5.77	1.46	0.74	1.09	1.27	0.04
*P values for main effects and interaction*							
Litter		0.129	0.404	0.819	0.083	0.597	0.064
Diet		0.536	0.337	0.508	0.690	0.286	0.003
Litter × diet		0.515	0.838	0.008	0.418	0.204	0.025

*SCFA* Short chain fatty acids; *C* Control diets; *TAG* Threonine, arginine and glutamine supplemented diets; *SED* Standard error of difference; Means within the same column with different superscripts differ at *P* < 0.05; Simple means represent 8 pens of pooled sampled from 2 birds sampled per pen

### Litter characteristics

At day 0, polymerase chain reaction (PCR) screens did not detect *Salmonella* spp., *Clostridium perfringens*, *Eimeria tenella* and *E. maxima* in either clean or reused litter. However, the 16S rRNA gene copy numbers of total bacteria per g of chicken litter were 2.08 and 8.13 copies per g of clean and reused litter, respectively. The initial pH of clean and reused litter was 5.76 and 8.09, respectively, whilst their moisture content was 10.79 and 12.49%, respectively.

The diet and litter treatment effects on final litter pH and moisture content are presented in Table 4. There were no significant interactions between diet and litter treatments for both parameters. Whilst diet treatment did not impact litter pH, placement of birds on reused litter resulted in significantly higher litter pH than their clean litter counterparts. In addition, both diet and litter treatments independently impacted litter moisture content, as the TAG and reused litter treatments reduced moisture content compared to the C and clean litter treatments, respectively.

**Table 4:** pH and moisture content of litter samples of 21-day-old broilers fed C or TAG diets and placed on either clean or reused litter at day-old

Litter	Diet	pH	Moisture, %
Clean	C	6.67	30.00
	TAG	6.57	27.36
Reused	C	7.35	25.10
	TAG	7.40	22.78
	SED	0.074	1.674
*Means for main effect of litter*			
Clean		6.62^a^	28.68^b^
Reused		7.38^b^	23.94^a^
	SED	0.052	1.184
*Means for main effect of diet*			
	C	7.01	27.55^b^
	TAG	6.99	25.07^a^
	SED	0.052	1.184
*P values for main effects and interaction*			
Litter		< 0.001	< 0.001
Diet		0.707	0.041
Litter × Diet		0.172	0.894

*C* Control; *TAG* Threonine, arginine, and glutamine supplemented diets; Means within the same column with different superscripts differ at *P* < 0.05; SED, standard error of difference; Simple means represent 8 pens per treatment

## Discussion

This study investigated the effects of Thr, Arg and Gln supplementation to IP formulated diets on performance, caecal microbiome and litter characteristics in the absence and presence of placement at reused litter to create two contrasting conditions in terms of microbiota exposure. We hypothesised that reused litter exposure at placement would affect the growth performance response to TAG supplementation. However, this hypothesis was rejected as TAG supplementation reduced performance for both clean and reused litter treatments. Supported by effects on caecal microbiome composition and SCFAs, collectively the data indicated that TAG supplementation may have resulted in excess protein over the IP-basis, which disadvantaged bird performance [33–35].

Here, birds placed on the reused litter had 6% greater BWG than those on clean litter. The use of reused litter has been shown to result in variable outcomes on performance, as it has been associated with penalised [36–39], similar [40, 41] or improved [42] performance relative to birds placed on clean litter. Such variable outcomes of using reused litter can be expected to arise from differences in litter characteristics, which are typically not reported in studies using reused litter, and include microbial composition, pH, moisture, and level of recycled nutrients from the previous flock. An early exposure of newly hatched chicks to reused litter facilitates the colonisation and cycling of microbiomes between gut and litter which accords with a probiotic or direct-fed microbial approach to improve intestinal microbiota and thus improved performance [32, 42]. In support of the positive effect of reused litter exposure on performance reported here, pathogens such as *C. perfringens*, *E. tenella* and *E. maxima* were not detected in the tested litter samples.

The reduced performance upon TAG supplementation may have a multi-factorial basis. Firstly, since diets were formulated to be isoenergetic, TAG supplementation as expected increased CP content and thus reduced the metabolisable energy to CP ratio. The latter may directly reduce the AA pool for protein deposition and uric acid synthesis as well as the supply of fat and carbohydrate to meet the energetic requirements of the birds [43]. Secondly, the AA availability in TAG supplemented birds might be lower than expected for growth due to increased proteolytic fermentation from excess AA, which has been shown to result in poorer intestinal health [33]. Around half of the undigested and unabsorbed protein is fermented by putrefactive caecal bacteria producing toxic compounds, i.e., amines, indoles, phenols, cresol and ammonia, which may impede performance [34, 35]. Thirdly, the AA imbalance arising from surplus AA in TAG supplemented birds might decrease the efficiency of utilisation of limiting AA for maintenance and protein deposition [44, 45]. This suggestion is supported by the reduced FI of TAG birds over C birds, as FI depression is one of the first manifestations of dietary AA imbalance in broilers [46, 47]. In addition, it has also been suggested that AA supplementation may reduce FI arising from the amino static hypothesis, in which free AA in plasma serve as a signal to an appetite-controlling mechanism [48–50].

The %G + C profile is used to indicate the relative abundance of bacteria with different DNA base compositions and hence allows detecting any putative alterations at the community level [51]. The most abundant bacteria observed in our study represent species with %G + C 40–55, such as *Lachnospiraceae* (Clostridial cluster IV) and *Lactobacilli*, which are known to dominate caecal microbiome composition of well-performing birds [52]. However, under-performing birds often have two peaks at <  ~ 37% and >  ~ 60 of %G + C instead of one peak at ~ 45%G + C [52]. Here, TAG supplementation resulted in a lower abundance of bacteria with %G + C 47–56, which indeed concurred with reduced growth performance over C birds, though there was no significant increase in the %G + C 20–30, which is often associated with the presence of pathogenic bacteria [53]. This suggests that a possible microbiological basis of the reduction in performance on TAG diets was most likely metabolic rather than pathogenic. Although birds on reused litter performed better and showed a greater proportion of propionic acid in their SCFA pool than those on clean litter, this did not concur with significant changes in microbial profile. Whilst there was some indication that the reused litter treatment indeed lowered bacterial abundance associated with %G + C 23–31 and increased bacterial abundance at %G + C 56–59 (Fig. 3), these did not reach statistical significance in this study.

Caecal SCFA analysed include the volatile fatty acids (VFA) acetate, propionate and butyrate, but also the non-volatile lactate, produced by gut microbiota as fermentation products from undigested nutrients [54]. The SCFA play a role in intestinal health, including the promotion of mucin production, blood flow, enterocytes growth and proliferation [55]. The VFAs mentioned are a valuable energy source for the host, especially butyrate being the preferred energy source for epithelial cells [56]. Furthermore, the increased proportion of propionic acid in the SCFA pool of birds on reused litter and fed C diets could indicate the presence of beneficial bacteria such as *Lactobacillus* spp.*,* which are known to have bacteriostatic or bactericidal properties against pathogenic microbes [52, 57]. This accords with the improved performance observed for birds on reused litter and fed C diet. The BCFA (iso-butyric, 2-methyl-butyric and iso*-*valeric) within the SCFA pool can only be produced from fermenting branched-chain AA, i.e., valine, leucine, and isoleucine. As such, variation in caecal BCFA levels may indicate variation in protein fermentation activity but also the flow of undigested protein into the caecum. Thus, elevated caecal BCFA could be indicative of reduced ileal crude protein (CP) digestibility, which would result in poorer growth performance [35, 58]. This is consistent with the elevated levels of iso-butyric acid in TAG- supplemented birds, being most pronounced on clean litter, and the reduced performance observed for those birds.

Litter pH and moisture content are some of the major determinants implicated in the survival and growth of litter pathogens [59]. Generally, litter pH ranges between 6.5 and 8.5, with negligible ammonia production below pH 7 [60, 61]. In the current study, diet treatment did not have a significant impact on the final litter pH, though reused litter pens had greater final pH levels than clean litter pens. However, temporal effects need to be considered, as pH for the clean litter pens increased from 5.76 at day 0 to 6.62 at day 21, whilst for the reused litter pens, pH decreased from 8.09 to 7.38. Both the difference at day 0 between clean and reused litter and the increase in pH for the clean litter pens over time can be attributed to the accumulation of excreta during the grow-out period, with elevated pH arising from protein degradation and ammonia production [62, 63]. However, whilst accumulation of excreta would also have occurred for the reused litter pens, the net reduction in pH overtime for these pens may be the consequence of continuing composting activity in situ as litter pH was observed to reduce over a period of 28 days for stored litter [64]. These data suggest that temporal rather than current variation in litter pH may better inform variation in performance, where the latter was greater for birds on reused litter compared to those on clean litter.

The final litter moisture for the TAG treatment was lower than that for the C treatment. This could be related to the reduced FI for the TAG birds, which would have concurred with reduced water intake as well as reduced total metabolites elimination with the excreta [65], both contributing to reduced water spillage and excretion. However, at similar levels of FI, reused litter pens also had lower final moisture content compared with clean litter pens. This suggests other reasons might also explain the variation in litter moisture and accords with previous studies [66, 67], where lower moisture content in litter used for multiple grow-outs (reused litter) was also observed. Reused litter has been found to have lower water activity and faster rate of excreta drying than clean litter, which might detriment the survival and growth of litter pathogens [67], and benefit performance.

## Conclusions

In this study, Thr, Arg and Gln supplementation to IP-based diets altered caecal microbial composition and enhanced proteolytic fermentation, indicative of excess protein leading to impaired performance. However, this study also supports the view that reused litter, particularly as assessed here in the absence of pathogenic bacteria, might benefit bird performance. The use of such litter accords with a probiotic or direct-fed microbial approach, combined with being a source of recycled nutrients.

## Materials and methods

### Bird management and experimental design

A total of 320 male Ross 308 broiler chickens were used in a 21-day experiment. Upon arrival (day 0), the birds were allocated to 32 floor pens (1.47 m × 0.94 m), separated through plastic-sheeted panels, with 10 birds per pen in a randomised complete block design. The temperature was set to 32 °C for the first 3 days and then was gradually reduced over a week until 25 °C was reached and maintained until day 21 as per breed guidelines. The light was provided for 23 h per day for the first week and then reduced to 18 h of light per day. Birds were provided *ad libitium* access to feed and water throughout the experiment, with feed offered as a meal. Birds were fed wheat-soyabean meal-based starter (0–11 days of age) and grower diets (11–21 days of age) with the control diets (see below) formulated to meet Ross 308 nutrient recommendations [68].

The experimental set up consisted of a 2 × 2 factorial arrangement of two diet treatments and two litter treatments (see below) with 8 pens per treatment combination within a complete randomised block arrangement.

### Diet treatments

The control (C) diet was formulated on an IP basis and supplemented with the synthetic AA to meet all eAA requirements on a digestible AA basis. The TAG treatment consisted of feeding the C diet with additional Thr and Arg at 25% above requirements and 1% Gln, as informed by previous studies [16, 69, 70]. For each phase, a common basal diet was prepared by including a 3% of corn starch for the C diets, against which the tested AA were included for the TAG diets. The TAG diets were therefore calculated to be isoenergetic but with varying CP levels. The ingredients and the calculated chemical compositions of the starter and grower diets are presented in Table 5.

**Table 5:** Feed ingredients and calculated chemical compositions (%) of the experimental starter (0–11 days) and grower (11–21 days) rations

	Starter rations		Grower rations	
	C	TAG	C	TAG
*Ingredients*				
Corn starch	3.00	1.44	3.00	1.51
Threonine	0.23	0.45	0.18	0.37
Arginine	0.10	0.44	0.05	0.35
Glutamine	0.00	1.00	0.00	1.00
Wheat	58.22	58.22	60.56	60.56
Soybean meal	31.59	31.59	28.50	28.50
Soya oil	2.20	2.20	3.50	3.50
Salt	0.05	0.05	0.05	0.05
Limestone	0.95	0.95	0.87	0.87
Dicalcium phosphate	1.85	1.85	1.65	1.65
Sodium bicarbonate	0.50	0.50	0.50	0.50
Lysine HCl	0.39	0.39	0.32	0.32
Methionine	0.24	0.24	0.21	0.21
Valine	0.09	0.09	0.06	0.06
Tryptophan	0.14	0.14	0.12	0.12
Isoleucine	0.05	0.05	0.03	0.03
Vitamin & mineral premix	0.40	0.40	0.40	0.40
*Calculated chemical composition*				
Crude protein %	22.47	24.51	21.01	22.95
AME MJ/kg	12.51	12.51	12.94	12.94
Calcium %	0.96	0.96	0.87	0.87
Phosphorous %	0.72	0.72	0.67	0.67
Available phosphorous %	0.48	0.48	0.44	0.44
Salt %	0.19	0.19	0.16	0.16
Sodium %	0.19	0.19	0.19	0.19
Chloride %	0.16	0.16	0.15	0.15
*Digestible essential amino acids %*				
Threonine	0.86	1.08	0.77	0.96
Arginine	1.37	1.71	1.23	1.53
Histidine	0.48	0.48	0.45	0.45
Isoleucine	0.86	0.86	0.78	0.78
Leucine	1.40	1.40	1.31	1.31
Lysine	1.28	1.28	1.15	1.15
Methionine	0.51	0.51	0.47	0.47
Cysteine	0.31	0.31	0.30	0.30
Tryptophan	0.20	0.20	0.19	0.19
Valine	0.96	0.96	0.87	0.87
Methionine + cysteine	0.82	0.82	0.77	0.77
Phenylalanine + tyrosine	1.58	1.58	1.48	1.48

*AME* Apparent metabolisable energy; *C* Control diets; *TAG* Threonine, arginine and glutamine supplemented diets; Vitamin and mineral premix provided (units kg^−1^ diets): Vit A, 16,000 IU; Vit D3, 3,000 IU; Vit E, 75 IU; Vit B1, 3 mg; Vit B2, 10 mg; Vit B6, 3 mg; Vit B12, 15 µg; Vit K3, 5 mg; Nicotinic acid 60 mg; Pantothenic acid 14.5 mg; Folic acid 1.5 mg; Biotin 275 µg; Choline chloride 250 mg; Iron 20 mg; Copper 10 mg; Manganese 100 mg; Cobalt 1 mg; Zinc 82 mg; Iodine 1 mg; Selenium 0.2 mg; Molybdenum 0.5 mg

### Litter treatments

For each diet treatment, half of the pens were supplied with all new wood shavings (clean litter) and the other half had 100% reused wood shavings litter (reused litter). The reused litter was derived from a previous 1152-bird broiler study (Ross 308) with no history of clinical diseases. The duration between litter collection and its reuse at the start of the current trial was 28 days, during which litter was untreated and stored in bags in an empty unheated shed.

### Sampling and data collection

#### Chemical analysis of diets

Experimental diets were analysed for dry matter (DM), neutral detergent fibre (NDF), acid detergent fibre (ADF), ether extract (EE), ether extract preceded by acid hydrolysis (AHEE), ash, starch, and total sugar (as sucrose) at Sciantec Analytical Services Ltd. (Cawood, UK) using standard protocols based upon Commission Regulation (EC) No. 152/2009. Analysis of CP and AA content, including tryptophan, were performed at Evonik Nutrition & Care GmbH (Hanau-Wolfgang, Germany). The CP was estimated using the Dumas method, and AA analysis was done by standard procedures [71] using an AA analyser (Biochrom 30 + , Cambridge, UK). Tryptophan was determined by high-performance liquid chromatography following preparation by hydrolysis. Gross energy (GE) was determined through an isoperibol bomb calorimeter system using benzoic acid as an internal standard (model 6200, Parr Instruments, Moline, Illinois, USA).

#### Growth performance

Growth performance parameters, i.e., BWG, FI, and FCR, were calculated from mean body weights (BWT) through bulk weighing and bird counting at pen level, weights of feed offered on days 0 and 11, and weights of feed refusals on days 11 and 21. The resulting BWG, FI and FCR were calculated for the entire growth period of days 0 to 21. Birds that were found dead or were culled were recorded for date, weighed and sent for post-mortem examination. BWG and total pen FI were corrected for mortality. FCR was calculated by dividing the average feed consumed per pen by the average weight gain of birds per pen. CPC was calculated by multiplying the average feed consumed by the dietary CP content and divided by the average weight gain of birds per pen as CPC = FI (kg) × CP content diet (g/kg)/BWG (g).

#### Caecal microbiome profile and short chain fatty acid analysis

At day 21, caecal digesta was collected in a sterile petri-dish from two randomly selected broilers per pen after being individually weighed, electrically stunned, and exsanguinated. Approximately 1 g of the pooled caecal content of the two birds was immediately preserved using BioFreeze™ sampling kits (Alimetrics Diagnostics Ltd., Espoo, Finland) following their recommended protocol pending analysis of total microbial community and SCFA using their in-house optimised and validated protocols [53].

The total microbial community was analysed using a culture-independent DNA-based method that was employed to determine the %G + C profile as described by [72]. The SCFA, which include acetic acid, propionic acid, butyric acid, the sum of the BCFA and lactic acid, were analysed using gas chromatography (Agilent Technologies, Santa Clara, CA, USA) as previously described [53].

#### Assessment of pathogens in reused litter

Representative litter samples for both clean and reused litter treatments were collected and analysed in triplicates at day 0 using sterilised gloves in self-sealed sterile plastic bags and kept at − 80 °C prior to analysis.

Litter samples were prepared for DNA extraction as previously described [73]. Briefly, 5 g of the collected litter sample was suspended in 30 mL of phosphate-buffered saline and then mixed for 5 min with an incubator shaker set at the maximum speed. Debris was removed by low-speed centrifugation (50 × *g* for 15 min at 4 °C), and the supernatant was collected in a sterile falcon tube. The bacteria were pelleted by high-speed centrifugation (3650 × *g* for 15 min at 4 °C) and resuspended in 1 mL of phosphate-buffered saline, whereas DNA was extracted using DNeasy PowerSoil Kit (Qiagen, United Kingdom) as per manufacturer instruction. The yield and quality of the DNA extracts were checked by NanoDrop 1000 spectrophotometer (Thermo Scientific, UK) at 260 nm.

DNA extracted from litter samples was used in PCR to test for the presence of *Salmonella* spp [74], *C. perfringens* [75–77], and *E. tenella* and *E. maxima* [78]. The specific genes, primer sequences, conditions, expected size of each amplicon, and PCR references are shown in Table 6. PCR reactions for amplification of the target genes were carried out in a final volume of 25 µL containing 1 × Q5^®^ Hot Start High-Fidelity Master Mix (New England Biolabs, UK), 200 nM of each primer (Table 6), 10 ng of DNA template and nuclease free water. The PCR cycling program consisted of an initial denaturation step at 98 °C (30 s), followed by 30 cycles of a 10 s denaturation step at 98 °C, a 30 s optimized annealing step at respective temperature (Table 6) and a 30 s elongation step at 72 °C, and a final extension step at 72 °C for 2 min before a 4 °C hold. The expected PCR amplification products were confirmed by agarose gel (1.5%) electrophoresis. Negative control template and positive control samples were included in each PCR screening. Positive controls for the bacteria targeted were prepared by isolating total DNA from pure cultures (Table 7). Positive controls for the *Eimeria* spp. were obtained isolating RNA from infected tissue as previously described (Table 7) [79].

**Table 6:** Primers for PCR and qPCR with PCR conditions

Pathogen	Target	Primer and probe sequences (5′–3)	Tm* (°C)	Amplicon size (bp)	Ref**
*Salmonella*	*ttr-4*	F:AGCTCAGACCAAAAGTGACCATC	66	94	[74]
		R:CTCACCAGGAGATTACAACATGG			
*C. perfringens*	16S rRNA	F:GGGGGTTTCAACACCTCC	63	170	[75]
		R:GCAAGGGATGTCAAGTGT			
	*CPα*	F:GCTAATGTTACTGCCGTTGA	60	324	[76]
		R:CCTCTGATACATCGTGTAAG			
	*NetB*	F:GCTGGTGCTGGAATAAATGC	65	383	[77]
		R:TCGCCATTGAGTAGTTTCCC			
*E. tenella*	*ITS1*	F:AATTTAGTCCATCGCAACCCTTG	65	279	[78]
		R:CGAGCGCTCTGCATACGACA			
*E. maxima*	*ITS1*	F:GTGGGACTGTGGTGATGGGG	65	205	[78]
		R:ACCAGCATGCGCTCACAACCC			
Total bacteria	*16S rRNA*	F:ACTCCTACGGGAGGCAGCAGT	60	194	[80]
		R:TATTACCGCGGCTGCTGGC			
		Probe:CGCGTGACCCTTATTGCTCCACA			

Tm*, optimised annealing temperature; Ref**, references

**Table 7:** Positive controls of the pathogens used in this study

Positive strain	Source
*Salmonella enterica subsp. enterica serotype Poona*	SRUC Veterinary Services
*C. perfringens* type A isolate MPRL 4739	SRUC Veterinary Services
*E. tenella*	RNA from *E. tenella* infected tissue [79]
*E. maxima*	RNA from, *E. maxima* infected tissue [79]

Quantification of the 16S rRNA gene was included as a proxy of total bacterial load and absolute quantification of the target was carried out based on Taqman probe chemistry as described previously [80]. Briefly, qPCR mixtures reactions were prepared in a final volume of 20 µL, containing a final concentration of ~ 7 ng per reaction of DNA template, 1X of Brilliant III Ultra-Fast qPCR Mastermix (Agilent Technologies, United States), containing 30 nM of freshly prepared reference dye (Agilent Technologies, United States) and 100 nM of each primer/ probe. Each reaction was carried out in triplicate in a 96-well plate, including non-template control and the standard curve. The latter was prepared via serial tenfold dilutions (10^7^ to 10^1^ gene copy numbers/reaction) of plasmid DNA containing the same target of this qPCR as an insert. Cycling conditions were set in a Stratagene MX3005P qPCR System (Agilent Technologies, United Kingdom) and were 95 °C (5 min), followed by 40 cycles of amplification at 95 °C (15 s) then 60 °C (30 s).

Absolute quantification was performed using the Stratagene MxPro Software (Agilent Technologies, United Kingdom) through fitting a linear regression model with log_10_ standard copy number [x] and standard threshold (CT) (y). The quality of the reactions was verified by analysing the slope of the standard curve regression R^2^ and efficiency calculation. The copy number calculated from the standard curve represented copies per µL of DNA extract. These values were log_10_-transformed and multiplied by 20 to obtain the 16S rRNA gene copy numbers per g of chicken litter [80].

### Litter pH and moisture analysis

At day 0, representative litter samples for both clean and reused litter treatments were collected using sterile gloves and self-sealed plastic bags. At day 21, representative litter samples were collected from each pen from the four pen corners and the middle (around the feeders and the drinkers) using sterile gloves and self-sealed plastic bags. The collected litter samples were kept at − 80 °C freezer prior to analysis. Litter pH was determined by placing 10 g of each litter sample into 90 mL of distilled water and mixing for 10–15 min. pH was then measured using a pH meter (Fisher Scientific accumet AE150 pH Benchtop Meter) after calibration with pH 4, 7 and 10 buffers. Litter moisture content was also analysed in duplicates by placing 10 g of each litter sample onto tared aluminium drying dishes in a drying oven at 100 °C for 24 h. Samples were removed from the oven, weighed, returned to the oven for 1 h and weighed again to confirm no further weight loss. Litter moisture was then calculated from the difference in sample start and end weight.

### Statistical analysis

Data were subjected to analysis of variance (ANOVA) using a GenStat 16 statistical software package (IACR, Rothamstead, Hertfordshire, UK). The data were analysed through a 2 × 2 factorial analysis of variance for diet treatments (C vs. TAG), litter treatments (clean vs. reused) and their interaction, using pen location as a block, day 0 BWT as a covariate for day 21 BWT and the pen of 10 chickens as the experimental unit. Data were checked for normality by examining residuals, histograms and box plots, and none required transformation prior to statistical analysis. Effects at *P* < 0.05 and *P* < 0.10 were considered significant and trends, respectively. Means were separated using Tukey's honest significance test.

## Data Availability

The datasets generated during and/or analysed during the current study are available from the corresponding author upon reasonable request.

## Abbreviations

AA: Amino acids
IP: Ideal protein
Thr: Threonine
Arg: Arginine
Gln: Glutamine
CP: Crude protein
BWT: Body weight
BWG: Body weight gain
FI: Feed intake
FCR: Feed conversion ratio
CPC: Crude protein conversion
SCFA: Short chain fatty acids
BCFA: Branched-chain fatty acids
%G + C: % Guanine + cytosine
